# Do financial performance and firm’s value affect the quality of corporate social responsibility disclosure: Moderating role of chief executive officer’s power in China

**DOI:** 10.3389/fpsyg.2022.925323

**Published:** 2022-08-18

**Authors:** Cao Na, Gaoliang Tian, Fawad Rauf, Khwaja Naveed

**Affiliations:** ^1^School of Management, Xi’an Jiaotong University, Xi’an, China; ^2^Centre for Management and Commerce, University of Swat, Swat, Pakistan; ^3^Faculty of Management Sciences, Riphah International University, Islamabad, Pakistan

**Keywords:** financial performance, corporate social responsibility disclosure, firm value, CEO power, agency theory

## Abstract

This paper investigates the correlation between the quality of corporate social responsibility disclosure (CSRD) and financial performance (FP). It also investigates the moderating role of chief executive officer power (CEOP) in the relationship between the quality of CSR disclosure and firm value (FV) in Chinese listed companies. The evidential research used the up-to-date sample (3, 248) of unbalanced findings for the period of 2014–2020, from the registered Chinese firms in the Shenzhen and Shanghai Stock Exchanges as samples for the study. As a starting point technique, the STATA 15 has been used to test pooled ordinary least squares (OLS) regression on a sample of Chinese listed companies. We use 1-year lagged regression and two SLS regressions to monitor the potential endogeneity problem. The imbalanced data set was received from the China Stock Market and Accounting Research (CSMAR) web page, which is the most significant source of information for Chinese publicly listed firms. Data on CSR information items and media reporting are compiled manually. The findings of the study revealed that there are positive FP consequences for the companies engaged in the quality of CSR disclosure. We also report that higher CEO power negatively enhances the quality of CSR disclosure effect on the FP of FV. The research investigates the impact of CSR disclosure and FP by presenting evidence of the moderating role of CEO power. Therefore, it is suggested that a higher law for CSR engagement and disclosure be implemented in China, and robust measures for the implementation of CEO power, although there are financial advantages to be gained. A key relevance to the empirical quality of CSR disclosure research can be recognized as the moderating role of CEO power in the quality of CSR disclosure, FP, and FV in the context of Chinese study. The findings are robust with the use of an instrumental variable method.

## Introduction

Corporate social responsibility (CSR) was “invented” in South Africa as a goodwill activity. The concept was promulgated as a term in the West primarily ascribed to Bowen and Johnson (1953). The United Nations (UN) then launched an environmentalist strategy in 1972 to urge corporations and communities to take action on major global challenges, such as inequality, human rights, and the environment. Following that, in 1987, the World Commission on Environment and Development produced a report on environment protection in which the phrase “ecological sustainability” was used for the first time. In 2015, the United Nations published its Goals of Sustainable development (UNSDG). The European Union (EU) agreed to these development goals and took the lead in formulating plans to attain them by 2030 (Qureshi et al., 2020). CSR, on the other hand, is a relatively new idea in China compared to the West. Although China’s economy is becoming increasingly relevant globally, CSR practices are, nevertheless, in their infancy (Zhang et al., 2019; Ma and Bu, 2021). China’s standards, attitudes, and working environment differ from those in the West, and these variations have an impact on CSR conception and activities (Wang and Juslin, 2009). Many publicly traded companies, for example, are not obligated to release CSR reports (Zhou, 2019). Global governance metrics show that China’s governance level is low when compared to the United States and developed European economies (Hewitt et al., 2021).

Referring to the vagueness of theoretical framework and analysis, according to Hanlon and Fleming (2009), the conception of CSR is vague from its beginnings and turned into a farce in the twenty first century. According to Hensel (Tan et al., 2016) (p. 68), proving the existence of the casual link between perceived phenomena requires more than their coexistence identification. The organizational researchers have a great number of variables in disposition, between which they can search for statistical relations (Mitchell et al., 1997; Liu and Jiraporn, 2010). Unfortunately, the field of organization studies faces dualism simultaneously: lack and excess of theories (Tan et al., 2016, p. 79–102). As a result, the theory of organization inquires about many theoretic concepts, implementing different methods (Chang et al., 2010). Hence, an organization researcher almost always is able to find the theoretical perspective, which will allow to justify the existence of observed correlation. Still, this justification would be rather weak and easy to undermine in the context of other theoretical approaches. A good illustration of mentioned situation is the debate on the positive impact of internationalization on the outcomes of enterprises. The protagonists try to find and justify this hypothesis by implementing different theories and explanations, while the others refute that (Chung et al., 2018; Hu et al., 2018). The no-existence of homogeneous theory is also seen as a problem in establishing the direction of cause-and-effect relation (Hui and Matsunaga, 2015; Singh et al., 2017). Moreover, many organizational variables are related bilaterally or create a feedback loop (Jamali, 2008). The higher motivation level determines effectiveness, which results in profits and, as a consequence, higher salaries. This reward further increases the level of motivation (Fanelli and Misangyi, 2006). It is very complicated to differentiate between the cause and the effect, i.e., the level of corporate social responsibility correlates with financial outcomes, but what is the direction of this relationship? Whether the corporations achieve better results as a consequence of implementing CSR or corporations with better outcomes are able to finance CSR activities (Reverte, 2009)? Answering the question of the direction of the relationship is frequently dependent on an arbitrary researcher’s choice (Hui and Matsunaga, 2015).

These practices have been assumed to be an agency cost on the part of firms and administrators (Li et al., 2016). In this situation, the CEO with power was thought of suspiciously as a tool for the covering of their payment, seeking behavior amidst their authority-based operating potential of the disclosure policy and material transparency. On the contrary, Tan et al. (2016) discard the agency cost based on a speculative hypothesis. However, the outcomes in this domain are diverse, and the literature does not have a definitive answer (Liu and Jiraporn, 2010; Tan et al., 2016).

Corporate societal practices are perceived in different ways by different administration subgroups–stakeholders with respect to their relative legitimacy and closeness. For example, a firm’s social action for a local community where the firm works will be valued differently by proximal teams and distant shareholders (Mitchell et al., 1997). Chang et al. (2010) describe the process of excellence in disclosure related to CEO compensation. They claim that delivering high qualitative information allows executives to have a good view of the firm’s underlying economic and strategic climate. Using comprehensive information, executives of the companies can easily scan the environment where they are operated, since parallel knowledge and information help them in building successful strategic and organizational decisions (Chang et al., 2010). Consequently, it enables them to analyze and compare benefits and costs related to consumers’ choices and environmental disclosures. Therefore, we investigate the role of CEO control, encouraged by the comparative payment of the CEO, in the correlation between reporting of ESG and firm valuation.

In this study, we use a robust CSR reporting proxy and a sufficient sample size to check whether CEO power has any effect on the relationship between firm values (FVs) and corporate social responsibility to confirm the connection between FVs and CSR. Furthermore, by analyzing the essence of the CEO in CSR transparency, we aim to explore the initial drivers of the association. While targeting this objective, we suggest disseminating previous tests of agency theory to clarify how the success of an organization is associated with the regulation of CEO power in the quality of CSR disclosure efficiency. Most of the research has tested the connection between a company’s values and CSR (Hui and Matsunaga, 2015; Singh et al., 2017; Chung et al., 2018). However, what has not been addressed is the moderating role of CEO power between the associations. Therefore, this research is an endeavor to fill the gap and contributes to an existing body of research.

A selection of research has found that a chief executive officer has the power to manipulate transparency policies. Song et al. (2020) offer proof of the motivation of the chief executive officer to monitor the details issued to the council. Also, a study by Goldman and Slezak (2006) considers the capacity of the CEO to stimulate knowledge disclosure. From the moment where the quality of disclosure represents the ability of executives to consider the underlying business landscape and to predict potential performance effectively, better exposure quality could suggest the Capability of the CEO to improve the FV (Hui and Matsunaga, 2015). The CEO’s efforts around the CSR acknowledgment can be a primary indicator of the quality of transparency at the heart of the executive committee. Therefore, we suggest that higher business expenses, induced by CSR transparency, would be higher in the occurrence of more CEO Control, as customers will potentially perceive the signaling impact of CSRD in terms of responsibility.

Furthermore, as per the principle of investor management (Jamali, 2008), managers stabilize different knowledge requirements of many stakeholders. However, their benefits and returns for all the stakeholders are not equal due to the specified objectives and goals of the firm (Fanelli and Misangyi, 2006). Different companies seek various possibilities from their creditors and have updated systems of priorities. Managers will offer additional scrutiny to their strains in companies with extreme investors and show more CSR results, regardless of the degree of company results. Chau and Gray (2010) propose that the mixed outcomes of the association between competitiveness and CSR disclosure could be underlined here by shifts in cultural and administrative problems between countries (Reverte, 2009).

Van der Laan Smith et al. (2005) find explorative evidence for Norwegian, Danish, and US businesses that control CSR disclosure through cultural and official influences. China has exclusive cultural and institutional geographies that influence CSR management motivations and objectives in an effective process, and the decision of managers to report CSR details from now on. However, from the viewpoint of stakeholder management, investor pre-eminence (the belief that there are only two businesses making returns for investors) tends to dominate. The shareholders and firms have fewer incentives for CSR (Van der Laan Smith et al., 2005). China has already declared many CSR stimulus programs, and the demand on Chinese firms from investors to prove CSR is relatively small, and there is little potential for investors (Lin, 2010). Risk management and stakeholder theory imply that fair capital investment and wealth resulting from investor association are created by CSR (Lin, 2010).

This increased wealth generates company goodwill, which can be a source of security as a guarantee in low-income regions. This hence boosts poor investors’ evaluation (Godfrey, 2005). The stakeholder theory of social control rights that CSR has a confident relationship with FP (Voinea et al., 2022). The agency theory postulates that a high-powered chief executive officer will generate a fight between management and owners who are fundamental to the issue of the agency on the contradictory thinking, and corporate and managerial thinking indicates that strongly managed CEOs have extra benefits and are less costly (Sah and Stiglitz, 1986). The relationship between chief executive officer pay and FP (Bebchuk and Fried, 2003) has been discussed in administrative control theory. The principle of stuff rights indicates that the structure of possession has a favorable relation with FP (Ceptureanu et al., 2017).

Similarly, several explorations have been carried out, particularly within economics and FV, relationships, and impact among CSR and communal company results. The findings are still inconclusive and appear inconclusive because of certain characteristics (Griffin and Mahon, 1997), and as a result, the fragmented outcome opens doors for future researchers to test the correlation between CSR and corporate financial performance (FP). Mishra and Suar (2010) have created a lot of interest among scholars. The association between CSR and FP has been identified in most research and is generally resounding. Previous research has shown that there is a strong relationship between the quality of CSR disclosure, FP, and the role of CEO power (Barnett and Salomon, 2006; Hui and Matsunaga, 2015). A negative association was also conveyed in several other studies (Wright and Ferris, 1997), while some studies have acknowledged no correlation between CSR and corporate FP (Barnett and Salomon, 2006).

Irrespective of their contrasting views toward the duty of managers, the view of owners and the view of creditors are individually universal (Donaldson and Preston, 1995). The interactions between managers’ perceptions and owners are difficult to match. But, a developing research stream demonstrates that there is an immense variation in the motivations of managers to respond to the welfare of investors and/or investors, and hence we concentrate on a primary motivation “cupidity” for managers in this research. For our research, this is a very important motivation, since we believe that top managers and executives’ cupidity disrupts the normative perceptions of both the investor and the perspective of the investors (Mannor et al., 2016; Gupta et al., 2020). Takacs Haynes et al. (2017) have also been convinced that corporate avarice adversely pushes the rights of shareholders as a show of manager opportunism. Instead of caring for the growth of shareholder equity, selfish chief executive officers direct additional capital from their businesses to personal interests, which outcome in a decrease in corporate profits (Takacs Haynes et al., 2017). We strengthen this result by offering that arrogance, in the best financial interest of other clients, would also adversely influence the ability of managers to succeed. More explicitly, we expect cupidity chief executive officers to minimize corporate social responsibility expenditures and lead their companies to the adverse effects of full shocks, actions that therefore go beyond what is expected according to the investor’s point of view.

To achieve the study’s goal, we gathered a panel of 3,248 publicly traded Chinese companies from 2014 to 2020. To the best of the author’s knowledge, this study is the first attempt to explore the association between the market value of firms and 11 unique dimensions of CSR in China. The study contributes to the body of knowledge: First, overall CSR disclosure has a negative influence on the FV of Chinese enterprises, implying a win-lose position. Second, environmental management harms the market value of Chinese enterprises, whereas employee performance has a favorable impact. Third, contrary to the overall findings, there is a positive association between FP and the market firm’s value of sensitive industry enterprises, implying a win-win situation. Fourth, prominent Chinese CEOs’ power integrates CSR investments with their companies’ long-term goals, creating a win-win situation. The study’s findings are unaffected by industry bias when evaluated to other CSR proxies.

The rest of the paper is organized as follows: Section 2 discusses theory and hypotheses in the purview of extant literature, Section 3 presents data and methodology, Section 4 illuminates the empirical results, and lastly, Section 5 concludes the findings of the research study.

## Theoretical framework and hypothesis development

This study investigates the correlation of the firm’s FP and firm’s value with the quality of CSRD under the moderating role of CEO power in China. The theory we have inculcated here is the stakeholder-agency theory. The entrenchment of the CEOs in Chinese firms makes it imperative to address the correlation under this perspective (Cha and Rajadhyaksha, 2021), as the extant literature has highlighted the difference in CSRD quality across different contexts (Rauf et al., 2021b).

Stakeholder–agency theory puts forward the compatibility between the agency and stakeholder theory in the midst of the harmful contest between the principal (stakeholders) and the agents (CEO) (Shankman, 1999; Thosuwanchot, 2021). When an executive holds a powerful position, he or she prioritizes his/her perceived efficiency, and the probability of agency issues like data irregularity and misrepresentation, and moral hazards arise (Fama and Jensen, 1983), which entice risks of information asymmetry (Lemma et al., 2021). This ambitiousness beyond the stakeholders’ interest aggravates the agency issues. This agency problem is further exacerbated when a decision maker like a CEO with no concatenation of financial decision hazards has entrenchment in the firm in the shape of power. This could result in the incomplete and/or erroneous reporting of the CSR while potentially harming the interests of the stakeholders. In the case of the non-entrenchment of the CEO, the regulators could check the stakeholders’ value-destroying activities. The agency theory hence argues for the non-entrenchment of the CEOs to leverage a good-quality CSRD (Razzaq and Niazi, 2018).

### Corporate social responsibility disclosure and firm financial performance

For more than three decades, several theoretical and empirical studies have been carried out to address the potential connection between CSR disclosure and FP (Marom, 2006; Sial et al., 2018a; Voinea et al., 2022). Among all those, studies by Anser et al. (2018) and Yang et al. (2019) are widely regarded as pioneering work in this area, focusing on the relationship between corporate social responsibility and FP (Anser et al., 2018; Yang et al., 2019; Awaysheh et al., 2020). This research also evaluates the direction, resilience, and causality of the connection, generating both preliminary and conflicting results. Many studies have examined the relationships between CSR disclosure and FP in the existing industry sector (Arayssi et al., 2016; Cheng et al., 2016; Pham and Tran, 2020).

The relationship between disclosure of corporate governance and FP results, such as liquidity, ownership, organization size and age, leverage, productivity, solvency, and assets turnover, was investigated by many researchers (Almajali et al., 2012; Hermuningsih et al., 2020). For instance, Ghelli (2013) conducted a study including 3,248 Fortune 500 businesses and the scientists gathered data on financial reporting and CSR transparency from the CSMAR database. The results showed the strong and relevant cause–effect relationship between CSR disclosure with business success (ROA) and firm valuation (Tobin’s Q) and CEO power’s significant negative cause–effect relationship. Nevertheless, financial success (ROE) was found to be strongly correlated with the disclosure of CSR.

Haniffa and Cooke (2005) find that decent-performing businesses contribute to more CSR disclosure details to legitimize their truth. The positive relationship between financial results and notification of CSR is attributed to the management of possibilities and flexibility to distribute further corporate social responsibility activities to investors (Haniffa and Cooke, 2005). A good association was discovered between financial results and disclosures related to the case (Tagesson et al., 2009; Mughal et al., 2021). It was found that there is a positive correlation between corporate social responsibility disclosure (CSRD) and the firms’ financial results, provided the company has good economic standing and the business can manage the expense of CSRD.

Li et al. (2013) conclude that firms with a higher return on investment are regulated by CSR transparency of high quality. A constructive association between financial reporting and CSR transparency proposes that CSR obligations make it easier to report on investor expectations even though there is no considerable change in corporate social responsibility obligations and quality (credibility of worth and client’s expectation). Adding to this, older industries with continuous success lead to CSR behavior in which concern is paid to sustainability impact (Withisuphakorn and Jiraporn, 2016; González-Rodríguez and Díaz-Fernández, 2020). Our research supports the study of Li et al. (2016) by seeing a wider China business sample and by spanning a longer period from 2008 to 2015. We oppose a successful relationship between the financial results and CSRD among Chinese firms, based on the expense debate (Li et al., 2016; Voinea et al., 2022).

Hypothesis (H1): All the composite financial performance has a positive effect on CSR disclosure.

### Firm’s value and corporate social responsibility disclosure

Concerning social responsibility disclosure, firms try to disclose their social obligation’s fulfillment. More elaborating, it means corporate entities try to communicate to outsiders about their fulfillment of social obligations, by disclosing information about social activities. In addition to that, disclosures are meant to check on the rage of the people who view the corporate entity as only a tool for money minting (Gupta, 2011; Dhar et al., 2022). CSRD contributes to shareholders in measuring the essential value of a business over recognizing maintainable organization performance or liable liabilities, such as litigation, which balance “intangible assets” that can consequence from the absence of rule or execution like defending against ecological damages or the human rights desecrations.

This is because CSRD interconnects an organization’s performance to minimize risk, produce inducements to manage risk, and deliver data on an organization’s human capital, its clients, and society. CSRD could increase a company’s community status and thus long−term investor wealth (Faisal et al., 2020). According to the shareholder’s view, CSR performance must be impartially revealed as valuable: “data is gilded to the shareholder”(Ortas and Gallego-Álvarez, 2020). Considerable like economical reporting, CSRD would contain dependable and applicable data on real corporate social responsibility performance slightly than simply reckoning events that can decrease under corporate social responsibility. Reasonable demonstration stops companies from using CSRD as an advertising instrument to hide weak corporate social responsibility performance (Cho et al., 2015). If CSRD properly exists and is of the best quality, it can raise information and competence, and decrease transaction charges through data irregularities.

However, executives can use optional CSRD necessity resourcefully (Khan et al., 2022). The study proposes that they might have a positively prejudiced discernment of their corporate social responsibility actions (Cormier et al., 2004; Saz-Gil et al., 2020). Furthermore, companies with weaker corporate social responsibility performance, usually in the ecological dimension, report more CSRD information (García-Sánchez and Araújo-Bernardo, 2020), consuming positive language and smaller inevitability than well-performing companies (Cho et al., 2010). Thus, CSRD information can include positively prejudiced information when CSRD information is optional and when the administration prefers to cause–effect positive rather than negative information (Cho and Kim, 2012; Du and Yu, 2021). This unfairness can result in prediction errors and eventually in a sophisticated cost of capital. It is also recommended that the CSRD of companies with weaker corporate social responsibility performance consequences in developed forecast fault be slickened to companies with superior CSR performance (Dhar et al., 2022). Hence, shareholders might have problems processing CSRD data appropriately (Veprauskaitė and Adams, 2013). A higher quality of CSRD can propose larger corporate social responsibility performance, but the open experimental query remains as to whether shareholders trust the simple volume of CSRD or whether they measure corporate social responsibility performance with regard to its influence on essential business wealth (Buallay et al., 2020). So, we hypothesize that CSRD is positively related to business wealth and that the influence of business wealth effect on CSRD will be an influencer for the low−performing business organization than high corporate social responsibility performers.

Hypothesis (H2): *The relationship between firm values is positively associated with CSR disclosure.*

### Moderating role of chief executive officer power on firm’s financial performance, firm value, and corporate social responsibility disclosure

The potential influence of FV on the amount of CSRDs by limiting the observing capacity of a board is chief executive officer control. From multiple influences (Jackling and Johl, 2009), chief executive officer control, such as chief executive officer ownership, chief executive officer duality, family chief executive officer rank, and chief executive officer tenure. A strong chief executive officer could influence the management decisions (Lim and Chung, 2021), eventually minimizing the board’s effectiveness (Adomako et al., 2021). In comparison, administrators may engage in broader dialogue and debate under the guidance of a controlling chief executive officer and deliberate on a wide variety of opinions (Zahra and Pearce, 1989; Dabbebi et al., 2022). A chief executive officer who often headed the committee can have a larger influence on the committee (Chithambo et al., 2020), since the chair also sets the task for committee meetings and can also control the topics before the committee (Herawati and Bernawati, 2020). Similarly, chief executive officers who assist as chairs often have an effective impact on the placement of management seat candidates. This raises the probability of insufficient influence of new independent board appointees of management despite their independence. About this matter, Haniffa and Cooke (2002) aim to offer opposing advice. When the two distinct characteristics of the chief executive officer and chair are introduced by two different persons, the efficiency of monitoring checks and balances is strengthened (Haniffa and Cooke, 2002). However, the division between these two pieces is not obligatory on an ongoing basis, and many businesses are well managed and show ownership of the power board in circumstances where these characters have been individual. In businesses where a chief executive officer has the role of duality, the chief executive officer also has more control that will allow him/her to make a decision that does not consider investor interests (Saona et al., 2020). This will also result in a loss of focus and involvement in social or civic activities and thereby impair reports relevant to them. A chief executive officer who owns a majority of the company’s stock is impacted by management actions and would also balance his/her inducements (Chithambo et al., 2020). However, it has also been discussed that reduced ownership concentration will contribute to suitable entrenched managers (Shakri et al., 2021). An existing chief executive officer may govern the judgments of boards on business strategy and rules relating to administrative social behavior. Previous literature shows that executive ownership has a detrimental impact on voluntary CSR disclosures (Chau and Gray, 2010).

If a chief executive officer has retained his or her job for a prolonged period, the agency issues will continue to grow, since the service duration of the chief executive officer has been seen to raise formal authority (Chithambo et al., 2020) concluded entrenchment. An influential chief executive officer is likely to put less focus on the rewards of clients and will not be able to invest in societal events. A family chief executive officer (identified by friends and family) is often required to make choices that protect the family’s interests. Likewise, a family chief executive officer has a key role in recruiting members of the board and might appoint external managers based on associates. In comparison, the family chief executive officer tends to be less open to ordinary shareholders (Lu et al., 2021). Therefore, it is estimated that relative to CSR policies and behavior, family chief executive officers will be less involved than non-family chief executive officers. In short, a chief executive officer’s control is likely to be a result of control, the duality of the chief executive officer, service duration, and family status.

In contrast to those reports, we consider only two inquiries involving chief executive officer control as a CSR disclosure moderator-financial success association (Javeed and Lefen, 2019). Li K. et al. (2018) analyzed the impact of firm performance (Bloomberg ratings) on financial performance (ROA) and integrated 2,415 United Kingdom firm-year findings between 2004 and 2013. Chief executive officer influence became more evident in the favorable effect of CSR transparency on financial results (Li Y. et al., 2018; Javeed and Lefen, 2019). The correlation between investor participation (based on the Eth Vest database) and environmental efficiency has also been found to be correlated with three forms of chief executive officer authority (environmental data control, formal power over the management board, and position power over the top management) and suggested a moderator impact of all sorts on the environmental success of investor’s engagement connection (Walls and Berrone, 2017).

The evidence on the association between FV and CEO power is also diversified. Recent research on the voluntary release of financial detail records by CSR indicates that the authority of the CEO affects the consistency of CSR disclosure, and high-quality disclosure of CSR improves the value of the firm (Rashid et al., 2020; Xu et al., 2020). Nevertheless, there is no study exploring the role of the CEO in the disclosure of CSR, although Hui and Matsunaga (2015) offer a subjective indicator that CEO control takes responsibility for the relationship of firms with government investment. Our research ventures to inspect the role of CEO power in CSRD, specifically whether it affects the relationship between firm valuation and CSRD. Therefore, we recommend the hypothesis as follows:

Hypothesis (H3a) The power of the CEO negatively impacts the level of CSRD.

Hypothesis (H3b): CEO power negatively moderates the relationship between CSRD and the firm’s FP.

Hypothesis (H3c): The conclusion of FV on CSR disclosure is more noticeable when the firm negatively moderates CEO power.

## Data, measurement, and research methodology

### Sample area

We used data from China-listed companies on the Shanghai and Shenzhen Stock Exchanges in our analysis. All the study variables of the data were gathered from the Chinese Stock Market and Accounting Research (CSMAR) report. For the period 2014 to 2019, finally, after removing the findings with missed values, we landed at a final sample of 3,248 findings for the business year.

### Corporate social responsibility disclosure

As our dependent variable, we used CSRD. On the bases of the yearly reports of listed companies in China and the reports of CSR information released by the China Stock Market and Accounting Research (CSMAR) official websites. It has a specialist CSR measurement method that has been described as the most up-to-date and reliable dataset of adequate CSR information (Usman, 2020; Rauf et al., 2021b). The detailed CSR disclosure score published by CSMAR, ranging from 0 to 1, was also applied in our research. In line with the previous research, we used the CSR assessment model (Haniffa and Cooke, 2005; Sial et al., 2018b) Table 1 indicates the calculation of the CSR disclosure information.

**TABLE 1:** Index of corporate social responsibility disclosure.

S. no	Items of CSR disclosure	Scale
(1).	Mentioning GRI standards or not	1.0
(2).	Inclusion of shareholder benefits	1.0
(3).	Inclusion or exclusion of creditor interests	1.0
(4).	Inclusion or exclusion of employee interests	1.0
(5).	Inclusion or exclusion of supplier interests	1.0
(6).	Inclusion or exclusion of interests of clients and consumers	1.0
(7).	Inclusion or exclusion environment	1.0
(8).	Inclusion or exclusion of public and social welfare	1.0
(9).	Inclusion or exclusion CSR system development	1.0
(10).	Inclusion or exclusion secure production	1.0
(11).	Inclusion or exclusion deficiencies of the firm	1.0

A binary method is implemented through $\sum_{n\,\frac{x}{n}\times 100}^{1},$ where x equals 1 in case of reporting an item and otherwise 0, and *n* represents the number of all items.

### Financial performance

In our study, the independent variable is FP Measures. The market-based measures, like return on assets (ROA), are usually used in current studies (Sial et al., 2018a; Song et al., 2020); however, some scholars have contested its suitability in China due to stock market efficiency (Liu et al., 2014). In contrast, accounting-based constructs are considered more reliable (Guest, 2009). Thus, we use ROA as a measure of FP.

### Firm value

In our analysis, the value of the firm is another independent variable, which is Tobin Q. The marked-based Tobin Q calculation shows the shareholders’ forward-looking valuations. The value of the firm (Tobin Q) is the stock valuation of the company divided by the gross asset book value (Dushnitsky and Lenox, 2006; Manrique and Martí-Ballester, 2017). The stock valuation of the company is determined based on the market value of the share plus the book value of the debt, where the market price of the shares is calculated by calculating the gross equity by the current share price.

### Moderating role of chief executive officer power

We calculated chief executive officer power (CEOP) in a past analysis, where CEO power is the moderating component (Veprauskaitė and Adams, 2013). This study has used Executive compensation in replacement of CFO influence. The CEO power was determined as an annual CEO salary split by all rewards from the board members. To locate the companies with high CEO strength, we built a quartile of the distribution. Following the research by Li Y. et al. (2018), we assort 1 for the firms in the top quartile for high chief executive officer capacity at 1 and others at 0 (Li Y. et al., 2018).

### Estimating model

To inspect the main impact of financial efficiency and FV effects on CSRD, we estimated Eqs. (1, 2), in line with previous research (Li Y. et al., 2018; Javeed and Lefen, 2019; Zhou, 2019; Usman, 2020; Rauf et al., 2021a; Voinea et al., 2022), to study the effect of FP and value of the firm on the CSRD of the Chinese stock exchange-listed manufacturing firms. We estimated Eqs. (3, 4) (Hu et al., 2018) to check the moderating effect of CEO power on the relationship between FP and CSRD and the FV and CSRD as follows:


(1) $$C\,S\,R\,{\text{D}}_{\left(i,t\right)}=a+\beta_{1}\,F\,P+\sum_{i=1}^{N}\beta_{n}\,c\,o\,n\,t\,r\,o\,l\,s_{\left(i,t\right)}+{ℰ}_{\left(i,t\right)}\,\ldots$$

(2) $$C\,S\,R\,{\text{D}}_{\left(i,t\right)}=a+\beta_{2}\,F\,V\,\sum_{i=1}^{N}\beta_{n}\,c\,o\,n\,t\,r\,o\,l\,s_{\left(i,t\right)}+{ℰ}_{\left(i,t\right)}\,\ldots$$

(3) $$C\,S\,R\,{\text{D}}_{\left(i,t\right)}=a+\beta_{3}\,F\,P+\beta_{4}\,C\,E\,O\,P+\beta_{5}\,F\,P\,x\,C\,E\,O\,P$$

$$\;\;+\sum_{i=1}^{N}\beta_{n}\,c\,o\,n\,t\,r\,o\,l\,s_{\left(i,t\right)}+{ℰ}_{\left(i,t\right)}\,\ldots$$

(4) $$C\,S\,R\,{\text{D}}_{\left(i,t\right)}=a+\beta_{6}\,F\,V+\beta_{7}\,C\,E\,O\,P+\beta_{8}\,F\,V\,x\,C\,E\,O\,P$$

$$\;\;+\sum_{i=1}^{N}\beta_{n}\,c\,o\,n\,t\,r\,o\,l\,s_{\left(i,t\right)}+{ℰ}_{\left(i,t\right)}\,\ldots$$

### Control variable

Based on earlier studies, at the company level, firm size, independent directors, board size, asset growth, book-to-market ratio, board meeting, financial leverage, CEO duality, and state-owned enterprises were considered as controlled variables, assuming that they provide significant support on the influence of CSR disclosures. These variables are firm size (FS) (Kallmuenzer and Peters, 2018), independent director (ID) (Ma and Khanna, 2016), book-to-market ratio (BTMA) (Cakici et al., 2017), asset growth (AG) (Chang et al., 2014), board meeting (BM) (Liang et al., 2013), financial leverage (FL) (Dalci, 2018), CEO duality (CD) (Rauf et al., 2021b), and state-owned enterprises (SOEs) (Chung et al., 2018). Finally, for the real effect of industry, we introduced industry dummies to control which included year dummies. See Table 2 for more information.

**TABLE 2:** Description of variables.

Variables	Abbreviation	Description
Corporate social responsibility disclosure	CSRD	Indicator variable equaling 1 for a firm voluntarily issuing a social responsibility report, and 0 otherwise
Financial performance (ROA)	ROA	It depicts the return on assets
Financial performance (ROE)	ROE	It is total profit divided by the ratio of equity.
Firm value (Tobin Q)	FV	To determine the Tobin *Q*-value, the numerator is the capital market value, the denominator is the gross sum of the commodity per year.
CEO power	CEOP	If depicts the CEO incumbent appointment.
Firm size	FS	Complete assets of natural log.
Board size	BS	The entire number of directors on the panel
Independent director	ID	The number of independent directors is divided by total directors, multiplied by 100.
BTMA	BTMA	It is a variable concerning the book value over the market value of the equity of shareholders.
Asset growth	FG	It is the change in total assets.
Board meeting	BM	A total number of board meetings held in 1 year.
F_Leverage	FL	The proportion of total debt to total assets
CEO duality	CEOD	A dummy number that is equal to one of the CEOs acts as chairman, and 0.
State-owned enterprises	SOEs	If the state or government governs the company, a dichotomous vector equals 1,
Year and industry	YI	This dichotomous variable is meant to control the year-wise and industry-wise variations.

## Empirical finding

### Descriptive statistics

Table 2 depicts the descriptive statistics of all the relevant variables used in this analysis. The average value of CSR disclosure is recorded as 5.807. The average value of FP is 2.421. The average FV is 2.954. Furthermore, we can see that many CEOs in the Chinese business are also the company’s founders. The average CEO power value is 0.179, depicting that 84% of the corporations have split the powers of CEO and Chairman. The average company size is 23.19. The average board size is 9.483, and the number of independent directors is 49%. The average BTMA in China is 1.186. The average asset growth value is 0.166, the mean financial leverage value is around 0.509, Board meeting is 10.215 and CEO duality is 0.155. Finally, the mean value of SOEs is 0.59.

### Correlation matrix

Table 3 presents the correlation matrix. According to Gujarati (2009), there is a strong multicollinearity problem (Gujarati and Porter, 2009), where the Pearson’s correlation matrix coefficient regarding the independent variables is higher than 0.80. The correlation coefficient in our analysis is between 0.01 and 0.60. Table 3 indicates that any strong multicollinearity issue could not influence the outcomes. In comparison, the inflationary factor variance (VIF) did not reach the limit of 3, and the maximum association between the variables remained smaller than 0.80.

**TABLE 3:** Descriptive and correlation statistics.

Variables	Mean	SD	Min	Max	(1)	(2)	(3)	(4)	(5)	(6)	(7)	(8)	(9)	(10)	(11)	(12)	(13)	(14)
CSRDQ	5.807	2.479	1.000	8.000	1.0000													
FP (ROA)	2.421	1.996	−0.776	1.216	0.007*	1.0000												
FP (ROE)	2.564	1.783	−0.689	1.378	0.006*	0.008*	1.0000											
FV (Tobin,s Q)	2.954	4.387	0.096	33.270	0.01*	0.02*	0.014*	1.0000										
CEOP	0.179	0.384	0.676	0.416	−0.06*	0.01*	0.10*	−0.07*	1.0000									
FS	23.192	1.747	18.265	30.814	−0.02*	0.06*	−0.48*	0.03*	0.07*	1.0000								
BS	9.483	2.282	4.000	22.000	0.04*	0.04*	−0.16*	0.01*	0.23*	0.05*	1.0000							
ID	3.493	0. 839	1.000	8.000	−0.03*	0.03*	−0.15	0.01*	0.31*	0.48*	0.01*	1.0000						
BTMA	1.186	1.153	0.030	10.32	−0.02*	0.07*	−0.50*	−0.09*	0.60*	0.09*	0.15*	0.20*	1.0000					
AG	0.166	0.343	−0.828	10.88	−0.05*	−0.01*	0.11	0.10*	−0.01*	−0.02*	−0.02*	−0.05*	0.51*	1.0000				
BM	10.215	4.790	1.000	57.000	−0.06*	0.01*	−0.09*	−0.01*	0.20*	0.00	0.04*	0.16*	0.10*	0.08*	1.0000			
FL	0.509	0.214	0.007	1.344	−0.01*	0.09*	−0.50*	−0.10*	0.51*	0.11*	0.14*	0.51*	−0.03*	0.22*	0.09	1.0000		
CEOD	0.155	0.362	0.000	1.000	−0.05*	0.00	0.13*	0.03*	0.11*	−0.13*	−0.08*	−0.12*	0.10*	−0.00	−0.12*	−0.02*	1.0000	
SOEs	0.599	0.489	0.000	28.624	0.07*	−0.20*	0.19*	−0.23*	−0.07*	0.07*	0.08*	0.11*	0.31*	0.10	0.09*	0.02*	0.11*	1.0000

*Represents the level of significance at 10%, respectively.

## Results

The OLS regression findings for checking the hypothesis are displayed in Table 4 (H1). The direct influence of FP results on CSR disclosure is shown in Model 1, and the coefficient of ROA is significant (*t* = 0.013, *p* < 0.001). The outcome withholds (H1) fostering that aggregate FP is favorably correlated to the CSR disclosure, and our analysis is in line with prior research results (Choi et al., 2010; Li et al., 2013; Sial et al., 2018b; Voinea et al., 2022).

**TABLE 4:** Regression results.

Variables	Model 1	Model 2	Model 3	Model 4
CSRD Q	OLS	OLS	OLS	OLS
FP (ROA)	0.003*** (0.01)	–	0.039*** (0.01)	–
FV (Tobin,s Q)	–	0.016*** (8.72)	–	0.007*** (5.39)
CEOP	–	–	−0.444*** (−3.46)	−0.498*** (−2.954)
FP × CEOP	–	–	−0.012*** (−3.321)	–
FV × CEOP	–	–	–	−0.014*** (−2.75)
FS	−0.011 (−0.34)	−0.069 (−1.571)	0.026 (0.633)	0.017 (0.425)
BS	−0.074* (−2.18)	−0.057 (−1.63)	−0.076* (−2.26)	−0.065* (−1.95)
ID	0.087 (0.97)	0.078 (0.85)	0.093 (1.04)	0.070 (0.79)
BTMA	−0.067 (−1.25)	−0.010 (−0.18)	−0.05 (−1.02)	−0.050 (−0.94)
AG	−0.009 (−0.07)	−0.015 (−0.11)	−0.027 (−0.20)	−0.043 (−0.30)
BM	−0.016 (−1.91)	−0.027*** (−2.92)	−0.019** (−2.287)	−0.017* (−2.06)
FL	−0.323* (−1.21)	0.051 (0.19)	−0.2452 (−0.91)	−4.132( −0.49)
CEOD	−0.249* (−2.18)	−0.225* (−1.97)	−0.257* (−2.28)	−4.267* (−2.35)
SOEs	0.010** (0.03)	0.013* (0.07)	0.007 (0.30)	0.007 (0.30)
Constant	6.075*** (7.00)	6.963*** (7.72)	62.35*** (3.77)	5.133*** (5.63)
YI	Included	Included	Included	Included
R2	0.1618	0.1277	0.1314	0.1637

*, **, *** depict significance at 10, 5, and 1%, respectively. The values in parentheses are that of T-statistics. A detailed description of variables is given in table 1.

To examine hypothesis (H2), Model 2 expanded our study a step further to assess whether FV influences CSR disclosure. The FV coefficient (*t* = 8.72, *p* < 0.000) is also positively important, suggesting that FV importance is greater in CSR disclosure and encourages greater CSR disclosure (H2). Our observations are consistent with previous research results (Crisóstomo et al., 2011; Hu et al., 2018).

We developed Model 3 to test the hypothesis (H3). We added CEO power as a moderator variable to our Model 3. The coefficient of CEO power is also negatively significant with the CSR disclosure in Model 3 and supports (H3a) in Model 3 (*t* = −3.46, *p* < 0.001). When we implemented our full interaction models FP × CEOP, the coefficient is also negatively significant with the CSR disclosure in Model 3 and supports (H3b) (*t* = 3.32, *p* < 0.005). Our results confirm that CEO power acts as a quasi-moderator, as suggested by a previous study (Javeed and Lefen, 2019).

Model 4 was developed to test hypothesis (H4), and the moderating influence of CEOP on the main effect was investigated. The coefficient of the interaction between FV × CEOP was substantially negative with the quality of CSR disclosure, as the regression result indicated, in Model 4 (*t* = −2.75, *p* < 0.000) Our results are in line with prior research results (Velte, 2019).

### Robustness tests with fixed and random effects, an alternative measure of corporate financial performance

The fixed and random effects are applied to the regression to check the robustness. We also use return on equity (ROE) instead of ROA as another metric to re-measure financial efficiency to verify the robustness of our results in this research. As presented in Table 5, the results remain significant and in line with our previous results listed in Table 5. The methodological findings remain largely unchanged, suggesting that our hypothesis is solid.

**TABLE 5:** Results with fixed effects and random effects (robustness test).

Fixed effects random effects								
Variables	Model 1	Model 2	Model 3	Model 4	Model 5	Model 6	Model 7	Model 8
CSRD Q	OLS	OLS	OLS	OLS	OLS	OLS	OLS	OLS
FP (ROE)	0.006*** (0.01)	–	0.0158** (0.07)	−	0.034*** (0.01)	–	0.038*** (0.01)	–
FV (Tobin,s Q)	–	0.014*** (8.72)	–	0.006*** (5.37)	–	0.001*** (3.35)	–	0.003*** (3.18)
CEOP	–	–	−0.442*** (−3.46)	−0.498*** (−2.95)	–	–	−0.444*** (−3.46)	−0.4971*** (−2.96)
FP × CEOP	–	–	−0.01*** (−3.34)	–	–	–	−0.014*** (−3.34)	–
FV × CEOP	–	–	–	−0.013*** (−2.74)	–	–	–	0.017*** (2.79)
FS	−0.014 (−0.34)	−0.0783* (−1.85)	0.010 (0.268)	0.010 (0.26)	−0.031 (−0.27)	−0.084* (−1.82)	−0.004 (−0.05)	−0.006 (−0.05)
BS	−0.068* (−2.09)	−0.057* (−1.73)	−0.061* (−1.87)	−0.061* (−1.87)	−0.070 (−1.50)	−0.054 (−1.48)	−0.055* (−1.55)	−0.055* (−1.55)
ID	0.069 (0.78)	0.079 (0.88)	0.055 (0.626)	0.055 (0.62)	0.078 (0.69)	0.083 (0.87)	0.053 (0.52)	0.053 (0.52)
BTMA	−0.076 (−1.44)	−0.012 (−0.24)	−0.059 (−1.15)	−0.059 (−1.15)	−0.111 (−1.27)	−0.043 (−0.77)	−0.090 (−1.21)	−0.093 (−1.21)
AG	−0.004 (−0.04)	0.006 (0.05)	−0.026 (−0.20)	−0.026 (−0.20)	0.008 (0.06)	0.050 (0.39)	0.004 (0.03)	0.004 (0.03)
BM	−0.017* (−1.98)	−0.023*** (−3.06)	−0.017* (−2.04)	−0.017* (−2.04)	−0.018* (1.16)	−0.026*** (−2.67)	−0.017* (−1.17)	−0.016* (−1.17)
FL	−0.277 (−1.02)	0.052 (0.18)	−0.091 (−0.33)	−0.091 (−0.33)	−0.417 (−0.81)	−0.075 (−0.246)	0.230 − 0.64)	−0.230 (−0.64)
CEOD	−0.255* (−2.21)	−0.214* (−1.81)	−0.266* (−2.30)	−0.266* (−2.30)	−0.219* (−1.76)	−0.171* (−1.46)	−0.216* (−1.74)	−0.216* (−1.74)
SOEs	0.0154*** (0.003)	0.024*** (0.01)	0.006 (0.40)	0.006 (0.40)	0.014*** (0.00)	0.015** (0.04)	0.006 (0.35)	0.006 (0.35)
Constant	6.1578** *(7.214)	7.137***( 7.73)	5.293*** (5.76)	5.293*** (5.76)	6.157*** (7.21)	7.403*** (6.89)	5.493*** (5.76)	5.295*** (5.78)
YI	Included	Included	Included	Included	Included	Included	Included	Included
R2	0.1618	0.1277	0.1635	0.1639	0.16897	0.12525	0.163838	0.16377
Hausman test (Chi2)	−	−	−	−	10.78 *** (0.0045)	10.88 *** (0.0056)	10.99 *** (0.0059)	10.95 *** (0.0062)

*, **, *** depicts significance at 10, 5, and 1%, respectively. The values in parentheses are that of T-statistics. The detailed description of variables is given in table 1.

### Controlling the endogeneity issue

In Table 6, we use two distinct models to verify the endogeneity problem. First, as the FP requires time to impact the decisions on CSR transparency, 1-year lagged financial results are used in the (OLS) regression. The findings suggest that these results are the same as those reported in Table 3. Second, we use the 2-SLS regression model to discuss the probability of endogeneity concerns (Bruynseels and Cardinaels, 2014) while utilizing FV as an instrumental component. Models 5, 6, 7, and 8 of Table 6 indicate that the 2-SLS regression results have also been confirmed by our key findings, as depicted in Table 3.

**TABLE 6:** Regression results with one year lagged OLS and two-stage least square (2SLS).

Models	Lagged OLS				2SLS			
CSRDQ	Model 1	Model 2	Model 3	Model 4	Model 5	Model 6	Model 7	Model 8
FP (ROA)	0.003*** (0.01)	–−	0.036*** (0.01)	–−	0.003*** (0.01)	–	0.037*** (0.01)	–
FV (Tobin,s Q)	–	0.0126** (8.74)	−	0.005*** (5.37)	−	0.017*** (3.36)	−	0.002*** (3.18)
CEOP	–	–	−0.440*** (−3.41)	−0.496*** (-2.99)	−	–	−0.442*** (−3.46)	−0.499*** − 2.96)
FP × CEOP	–	−	−0.012*** (−3.33)	−	−	–	−0.012*** (−3.34)	–
FV × CEOP	–	−	−	−0.013*** (−2.73)	−	−	−	−0.017*** (−2.79)
FS	0.004 (0.10)	−0.063 (−1.47)	0.025 (0.61)	0.025 (0.61)	−0.031 (−0.27)	−0.084* (−1.82)	−0.004 (−0.05)	−0.004 (−0.05)
BS	−0.0553 (−1.62)	−0.047 (−1.41)	−0.049 (−1.50)	−0.049 (−1.50)	−0.070 (−1.50)	−0.054 (−1.48)	−0.055* (−1.55)	−0.055* (−1.55)
ID	0.0551 (0.60)	0.068 (0.74)	0.041 (0.46)	0.048 (0.46)	0.078 (0.69)	0.083 (0.87)	0.053 (0.52)	0.053 (0.52)
BTMA	−0.089 (−1.62)	−0.027 (−0.51)	−0.074 (−1.42)	−0.074 (−1.42)	−0.111 (−1.27)	−0.043 (−0.77)	−0.090 (−1.21)	−0.097 (−1.21)
AG	0.007 (0.06)	0.029 (0.22)	−0.009 (−0.07)	-0.009 (-0.07)	0.08 (0.06)	0.050 (0.39)	0.004 (0.03)	0.047 (0.03)
BM	−0.015* (−1.87)	−0.026*** (−2.93)	−0.016* (−1.86)	−0.016* (−1.85)	−0.016* (1.17)	−0.026*** (−2.67)	−0.017* (−1.17)	−0.016* (−1.17)
FL	−0.212 (−0.77)	0.097 (0.33)	−0.047 (−0.17)	−0.047 (−0.17)	−0.417 − 0.81)	−0.075 (−0.246)	0.230 (−0.64)	−0.230 (−0.64)
CEOD	−0.225 (−1.93)	−0.180 (−1.50)	−0.234* (−2.00)	−0.23* (−2.01)	−0.219* (−1.76)	−0.171* (−1.46)	−0.2162* (−1.74)	−0.2167* (−1.74)
SOEs	0.012** (0.020	0.015* (0.04)	0.006 (0.32)	0.012 (0.17)	0.014*** (0.05)	0.015* (0.045	0.006 (0.35)	0.006 (0.35)
Constant	5.518*** (6.285)	6.647*** (7.16)	4.777*** (0.19)	4.776*** (5.18)	6.157*** (7.213)	7.4035** (6.89)	5.493*** (5.761)	5.295*** (5.78)
YI	Included	Included	Included	Included	Included	Included	Included	Included
*R* ^2^	0.16403	0.12668	0.16534	0.16546	0.16891	0.12524	0.16383	0.16376

*, **, *** depict significance at 10, 5, and 1%, respectively. The values in parentheses are that of T-statistics. A detailed description of variables is given in Table 1.

## Conclusion and policy implications

This study investigated the impact of CEO power on the relationships between FP and CSRD quality and firms’ value and CSRD quality.

The findings depict a negative association between CEO power and relationships. In the midst of the low performance of Chinese CSR procedures (Liu and Jiraporn, 2010), the entrenched power of the CEOs could be one of the factors leading to it. The findings verify the premise of agency theory regarding CEO power[43]. CEO power has been termed as a double-edged blade having the potential to affect the CG dynamics in both directions (Ciasullo et al., 2017). We have shown that in cases of FP and CSRD quality and firms’ value and CSRD quality, it hinders the monitoring function of the corporate boards amid the entrenchment of CEO power. Moreover, the conflict of interest on part of the CEO arises from the perspective of agency theory due to their urge for control, which keeps accountability at stake, a construct of good CG.

In the light of the above-mentioned argument, the stakeholders’ confidence in the top management shatters. It is for this reason that the Securities Regulatory Commission of China (CSRC) has prohibited CEO duality, and therefore other forms of CEO entrenchment also need to be prohibited. Based on the findings of this study, we imply that the selection of more independent boards can cater to this issue.

Based on the agency theory perspective, we have shown an aggregate positive impact of FP and firms’ value on the quality of CSRD. Our findings imply that a firm is more potent to disclose its CSR reports with more quality when it has better FP, better value, and is not entrenched by the sole power of the CEO.

This research contributes to the body of literature on the role of the top management teams’ behavior by examining how the powerful CEOs make use of their resources in terms of entrenched power for CSRD quality in the context of the firm’s value and FP of firms. The findings foster that firms with entrenched CEOs use their power to cover their inefficiencies. However, the role of firms’ value and FP outweighs the agency problems while explaining the behavior of firms with entrenched power in China. This research also provides new evidence to the body of literature investigating the role of top management teams by examining the impact of their power, whether positive in terms of outcome in the quality of CSRD. In firms with good FP and FV, the CEOs have different incentives to respond to the call of CSRD and impact its quality. These findings and conclusions also shed light on whether the CEO‘s agency issues are ameliorated in the context of firm performance and FV.

### Limitations and future research

In terms of limitations, though our results indicate a favorable association between business value and CSR transparency, first, to assess company value, this analysis is controlled by FV metrics, such as Tobin Q. If it absorbs CSR disclosure or that increased standard of sustainability data contributes to higher business valuation or businesses with greater business value appear to put more resources on the CSR disclosure, upcoming research could be required to explore the causal effects. A substitute technique is to clarify the relation elements from an economic experimental perspective, outside the traditional econometric framework we follow along the line of a previous study (Ciasullo et al., 2017).

First, relevant CSRD, such as ecological and sustainability reports, reports on the security of staff and consumer rights, reports on the protection of interests of stockholders, and other CSR strategies, may be correlated to potential research, like analysis of content, such as the number of terms, phrases, subsections, or other techniques. Second, to assess the financial efficiency of a firm, this study is confined to FP metrics, such as ROE and ROA. To find the connection between business results and CSR reporting, the upcoming inquiry should add better business performance metrics.

## Data availability statement

The datasets presented in this study can be found in online repositories. The names of the repository/repositories and accession number(s) can be found in the article/supplementary material.

## Author contributions

FR and CN: conceptualization. GT: methodology and supervision. FR: software, formal analysis, investigation, resources, and visualization. FR, GT, CN, and KN: validation, and writing—original draft preparation and review and editing. GT and KN: data curation. CN and GT: project administration. All authors have read and agreed to the published version of the manuscript.
